# Tungsten Diselenide Top-gate Transistors with Multilayer Antimonene Electrodes: Gate Stacks and Epitaxially Grown 2D Material Heterostructures

**DOI:** 10.1038/s41598-020-63098-1

**Published:** 2020-04-06

**Authors:** Yu-Wei Zhang, Jun-Yan Li, Chao-Hsin Wu, Chiao-Yun Chang, Shu-Wei Chang, Min-Hsiung Shih, Shih-Yen Lin

**Affiliations:** 1 Graduate Institute of Electronics Engineering, National Taiwan University, No. 1, Sec. 4, Roosevelt Rd., Taipei, 10617 Taiwan; 2 Research Center for Applied Sciences, Academia Sinica, No. 128, Sec. 2, Academia Rd., Taipei, 11529 Taiwan

**Keywords:** Electrical and electronic engineering, Two-dimensional materials

## Abstract

We have demonstrated that with e-beam deposition of a thin Al_2_O_3_ layer before atomic layer deposition, a uniform Al_2_O_3_ film can be obtained on WSe_2_/sapphire samples. Device performances are observed for WSe_2_ top-gate transistors by using oxide stacks as the gate dielectric. By using thermal evaporation, epitaxially grown multilayer antimonene can be prepared on both MoS_2_ and WSe_2_ surfaces. With multilayer antimonene as the contact metal, a significant increase in drain currents and ON/OFF ratios is observed for the device, which indicates that high contact resistance between metal/2D material interfaces is a critical issue for 2D devices. The observation of multilayer antimonene grown on different 2D material surfaces has demonstrated less dependence on the substrate lattice constant of the unique van der Waals epitaxy for 2D materials. The results have also demonstrated that stacking 2D materials with different materials plays an important role in the practical applications of 2D devices.

## Introduction

With increasing demand for smaller electronic devices, <3 nm technology node has become a bottleneck in Si industries. Therefore, in addition to traditional Si or strained Si materials, people have started to turn their attention to 2D materials^1–5^. Unlike bulk materials, 2D materials can exhibit material characteristics in just a few atomic layers. The thickness is usually below 1 nm, which is advantageous for device fabrication in the nm range. One of the most studied 2D crystals in the last decade is graphene^6^. With its ultrahigh mobility value, researchers believe that the material can be used for next-generation high-speed electronics. However, the zero bandgap nature of this material has limited its applications in logic circuits. Thus, people have gradually turned their attention to other 2D materials, such as transition-metal dichalcogenides (TMDs). The major advantage of TMDs is that these materials have bandgaps, and transistors with high ON/OFF ratios can be fabricated on these materials^7^. However, their limited field-effect mobility values have raised another concern for practical applications. Recently, researchers have again moved their research focus to other group V 2D materials, such as phosphorene, which is also known as black phosphorus (BP)^8^. BP is expected to have high mobility values and a bandgap value of approximately 1.75 eV. However, its device application is hindered by the rapid degradation of bP under atmospheric conditions^9,10^. Compared with MoS_2_ transistors, enhanced transistor performances based on WSe_2_-graphene lateral heterostructures have been demonstrated in another publication^11^. High ON/OFF ratios up to 10^7^ and acceptable mobility values up to 84 cm2/V·s were observed for the device. The results demonstrated that selenide-based materials can be a promising 2D material for electronic device applications.

However, for the application of 2D materials in transistors, there are two major challenges. Although bottom-gate transistors are frequently adopted in the literature to demonstrate the unique characteristics of 2D materials, the major device architecture that is on the market is the top-gate transistor. Therefore, one of the challenges lies in the growth of high-quality dielectric layers on 2D material surfaces. Because there are no dangling bonds on 2D material surfaces, it is difficult to grow dielectric layers directly on 2D material surfaces. Buffer layers and special treatments may be required before dielectric layer growth^12,13^. The other challenge for 2D devices is the choice of metal contacts. Unlike traditional semiconductors such as Si or GaAs, it is difficult to obtain Ohmic contacts between conventional metals adopted for semiconductor devices and 2D materials. It has been proposed in previous publications that by using either graphene or crystallized thin indium (In) films as the contact metals, significant contact resistance reduction can be observed for 2D devices^11,14^. The results have demonstrated that one possible solution for the choice of contact metals for 2D devices may be conducting crystals. In this paper, we have demonstrated that with a predeposited thin Al_2_O_3_ layer using an e-beam evaporator, a uniform dielectric layer can be obtained on WSe_2_ surfaces after atomic layer deposition (ALD) of an additional Al_2_O_3_ layer. By using the oxide stacks as the gate dielectric, device performances are observed for the WSe_2_ top-gate transistors. By using epitaxially grown multilayer antimonene on WSe_2_ surfaces as the contact metal, significant increases in drain currents and ON/OFF ratios are observed.

## Results and Discussion

### Monolayer WSe_2_ growth by using chemical vapor deposition

**Figure 1:**
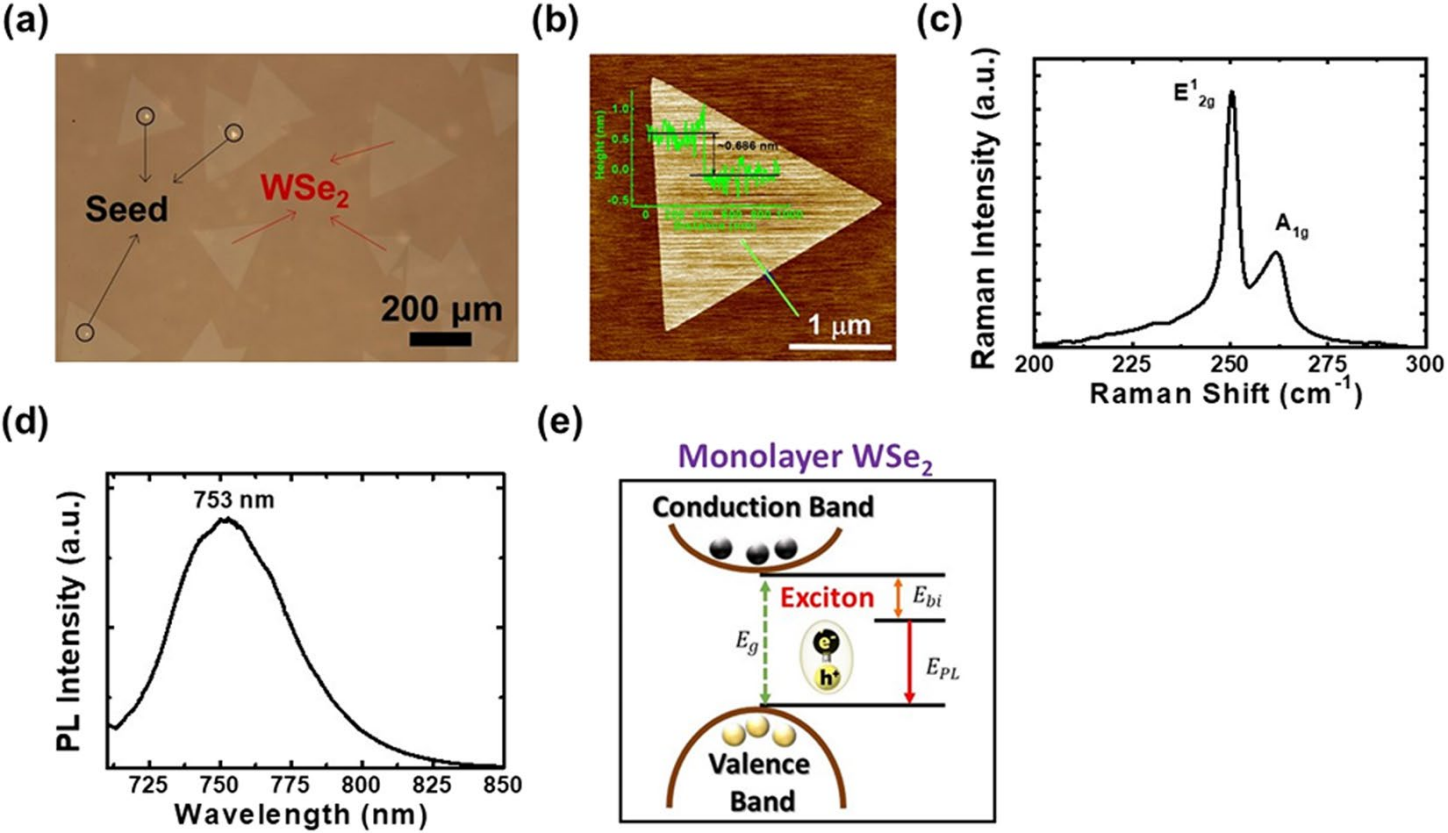
(**a**) Image of the WSe_2_ sample taken under an optical microscope. (**b**) Raman and (**c**) PL spectra of the sample. (**d**) Schematic diagram of the exciton transition energy for monolayer WSe_2_.

A picture of the WSe_2_ sample taken under an optical microscope is shown in Fig. 1(a). As shown in the figure, triangular WSe_2_ flakes with widths of 100–200 μm are obtained after the chemical vapor deposition (CVD) growth procedure. Also shown in the figure, smaller triangular WSe_2_ is present on the centers of some large WSe_2_ flakes, which suggests that a second or even third layer of WSe_2_ starts to grow from the center of the large flakes. The results demonstrate that the growth of WSe_2_ may initiate from a seed. The lateral growth rate of WSe_2_ on the sapphire substrate should be much faster than that on WSe_2_ surfaces. In this case, large WSe_2_ flakes would form before the second layer of WSe_2_ starts to grow on top of the large flakes. During CVD growth, H_2_ gas may act as the catalyst. Without H_2_ gas, the selenization procedure would not take place. To verify the thickness/layer numbers of the WSe_2_ flakes, the atomic force microscope (AFM) image of one WSe_2_ flake is shown in Fig. 1(b). The line profile on the edge of the WSe_2_ flake is also shown in the figure. As shown in the figure, the height of the flat WSe_2_ flake is around 0.686 nm, which is close to the thickness (0.7 nm) of mono-layer WSe_2_^15^. The results reveal that mono-layer WSe_2_ flakes are obtained by using CVD. The Raman spectrum of the sample is shown in Fig. 1(c). As shown in the figure, two characteristic Raman peaks corresponding to the lateral vibration mode ${{\rm{E}}}_{2{\rm{g}}}^{1}$ and the longitudinal vibration mode A_1g_ located at 250.3 and 261.8 cm^−1^, respectively, are observed. The observation of the two Raman peaks suggests that single-crystal WSe_2_ is obtained by using CVD^15^. The photoluminescence (PL) spectrum of the sample is shown in Fig. 1(d). Intense luminescence intensity located at 753 nm is observed. Because of the large exciton binding energy of WSe_2_, the exciton emission in monolayer WSe_2_ dominates the photoluminescence spectra at room temperature with emission peak at 1.625–1.660 eV (763–747 nm)^16^. The emission peak shown in Fig. 1(c) is at 753 nm (~1.65 eV), which is lower than the direct band gap value of ~1.89 eV for monolayer WSe_2_ on sapphire substrates due to the deduction of the exciton binding energy^17^. A schematic diagram of the exciton transition energy for monolayer WSe_2_ is shown in Fig. 1(e). The exciton transition energy (E_PL_) of monolayer WSe_2_ can be estimated by using the equation E_PL_ = E_g_ − E_bi_, where E_g_ and E_bi_ are the band gap energy and exciton binding energy of monolayer WSe_2_, respectively. The exciton binding energy thus obtained is 0.24 eV, which is consistent with previous results^17^. Since multilayer WSe_2_ turns into an indirect bandgap material, the intense PL intensity suggests that single-crystal and monolayer WSe_2_ with large flake sizes are obtained by using the CVD growth technique.

### Dielectric layer growth on WSe_2_ surfaces

**Figure 2:**
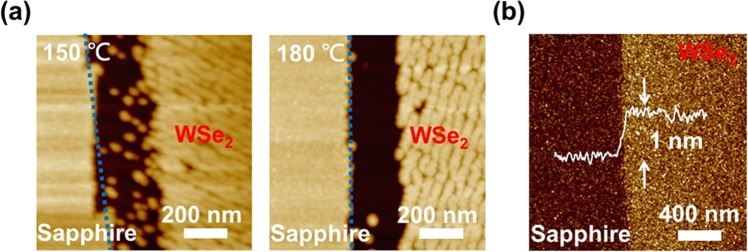
(**a**) AFM images of the samples with direct Al_2_O_3_ growth by using ALD at 150 and 180 °C. (**b**) AFM image of the sample with a 5-nm e-beam deposited Al_2_O_3_ layer before the 20-nm ALD-grown Al_2_O_3_ layer.

One major challenge for the fabrication of 2D material top-gate transistors is the growth of the dielectric layers. To investigate this phenomenon, 30-nm Al_2_O_3_ is grown directly on two WSe_2_/sapphire samples by using atomic layer deposition (ALD) at 150 and 180 °C. The atomic force microscopy images of the two samples are shown in Fig. 2(a). As shown in the figure, flat Al_2_O_3_ films can be grown uniformly on sapphire surfaces at the two different growth temperatures. However, grained Al_2_O_3_ films are observed on the WSe_2_ surfaces. On the edges of the WSe_2_ flakes, reduced Al_2_O_3_ grains are observed for the sample grown at 150 °C. The phenomenon became more pronounced as the growth temperature increased to 180 °C. Due to the lack of dangling bonds on 2D material surfaces, a non-uniform precursor distribution is obtained during the ALD growth procedure. In this case, grained Al_2_O_3_ instead of a flat oxide film is observed on 2D material surfaces. With increasing growth temperatures, the precursor on the 2D material edges is attracted to the sapphire substrate, and therefore, a region ~ 200 nm in width with no oxide coverage on the WSe_2_ edges is observed in the AFM image of the sample grown at 180 °C, as shown in Fig. 2(a). The two phenomena would both induce high gate leakage currents and result in device failure. To improve the quality of the dielectric layer, a thin 5-nm Al_2_O_3_ film is deposited by using an e-beam evaporator before the ALD growth of another 20-nm layer of Al_2_O_3_. The growth temperature for ALD is 180 °C. The AFM images of the sample are shown in Fig. 2(b). As shown in the figure, uniform Al_2_O_3_ films are observed on both the sapphire and WSe_2_ surfaces. The depth profile also shown in the figure reveals an ~ 1 nm step on the WSe_2_ edges with the sapphire substrates, which is close to the 0.7-nm layer thickness of monolayer WSe_2_. The results have demonstrated that with an additional 5-nm Al_2_O_3_ film deposited before ALD growth, uniform Al_2_O_3_ coverage can be obtained on 2D material surfaces. Unlike the absence of oxide growth on WSe_2_ edges, uniform Al_2_O_3_ growth across the interfaces is observed for the sample with the thin e-beam-deposited Al_2_O_3_ layer.

### Top-gate WSe_2_ transistors

**Figure 3:**
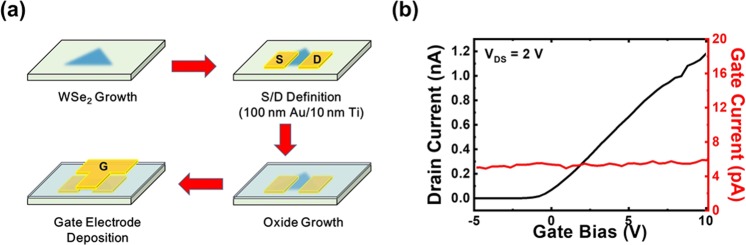
(**a**) The fabrication procedure and (**b**) the I_D_-V_GS_ curve of the WSe_2_ top-gate transistors with Au/Ti electrodes. The gate currents are also shown in (**b**).

The fabrication procedure of the WSe_2_ top-gate transistors is shown in Fig. 3(a). After WSe_2_ growth, 80 × 80 μm2 S/D electrodes with 100 nm Au/10 nm Ti are fabricated on the WSe_2_ surface following standard photolithography, thermal evaporation and metal lift-off procedures. After the S/D definitions, a 25-nm dielectric layer with a 5-nm e-beam-deposited Al_2_O_3_ layer before the 20-nm ALD-grown Al_2_O_3_ layer is prepared on top of the whole sample. Although the e-beam-deposited Al_2_O_3_ layers can provide better coverage of dielectric films, dielectric layers prepared by using ALD can provide more complete film growth with a proper choice of seeding layers on 2D material surfaces, which will lead to lower gate leakage currents. After that, the gate electrode with 100 nm Au/10 nm Ti is fabricated on the WSe_2_ channel. The I_D_-V_GS_ curve of the device at V_DS_ = 2 V is shown in Fig. 3(b). The gate currents of the device are also shown in the figure. With a low gate leakage current down to 10^−12^ A, it is demonstrated that the 5-nm e-beam-predeposited Al_2_O_3_ layer not only improved the film morphology but also depressed the gate leakage currents. Combining e-beam evaporation and ALD, we can avoid the problem of precursor distribution on 2D material surfaces with the help of physically deposited thin oxide layers (e-beam) and still obtain a flat dielectric layer through a chemical growth technique (ALD). In this case, n-channel transistor performances can be observed for the WSe_2_ top-gate transistors. However, the drain currents of the device are relatively low, which would result in a low ON/OFF ratio of 5 × 10^3^. There are several possible mechanisms responsible for this phenomenon. Since the channel is only monolayer WSe_2_, although the e-beam-predeposited Al_2_O_3_ layer helps establish a working gate dielectric, the 2D material channel may still be damaged during the oxide deposition procedure. Thus, 2D material heterostructures may help to prevent the channel from the significant influence of the oxide interface^18^. Further investigation is still required in the future. The other possible mechanism responsible for the low drain currents is the high contact resistance on metal/2D material interfaces.

### van der Waals epitaxy of antimonene on WSe_2_ surfaces

**Figure 4:**
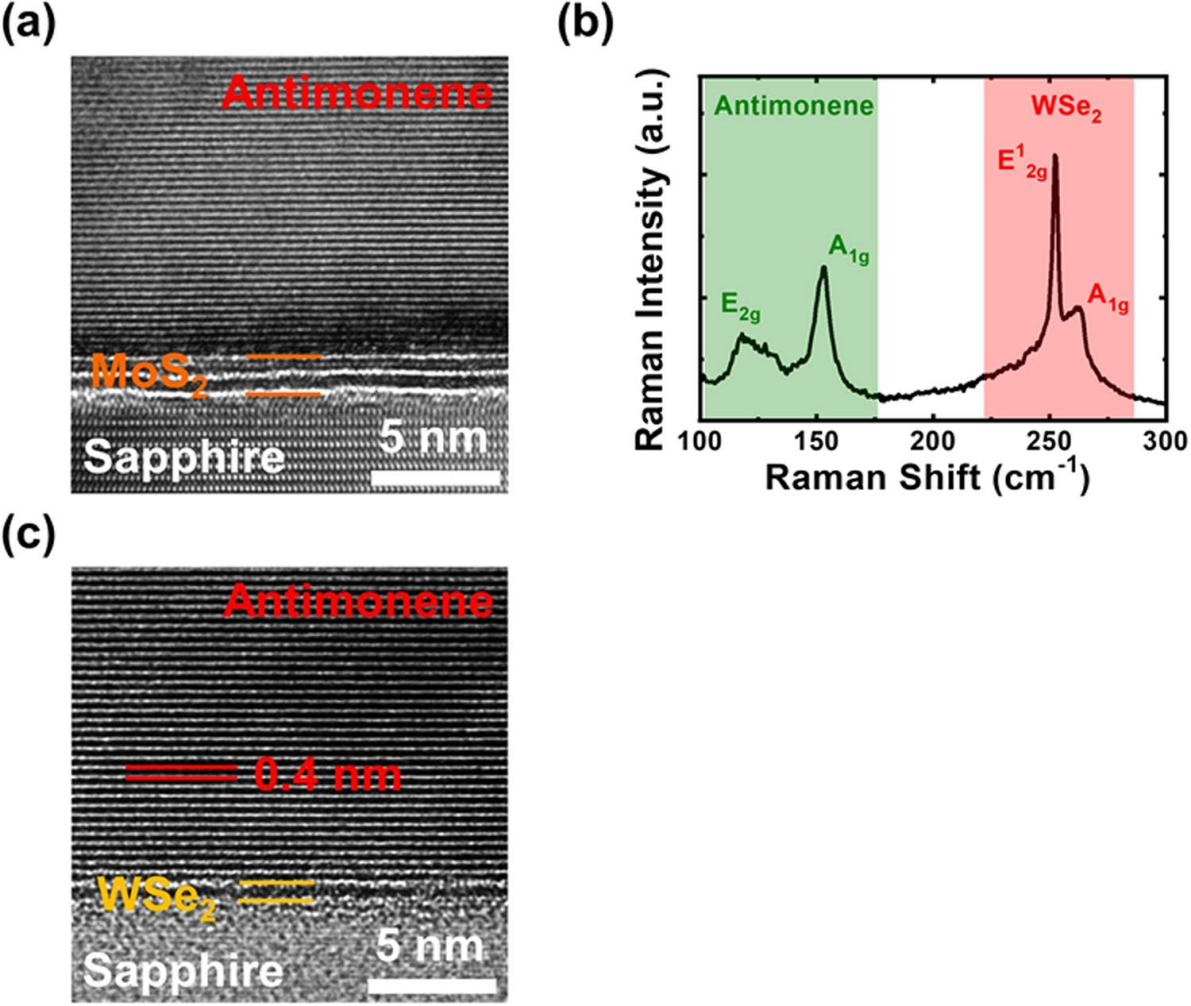
(**a**) Cross-sectional HRTEM image of the sample with 50-nm antimony deposited on the MoS_2_ surface by using thermal evaporation. (**b**) The Raman spectrum and (**c**) the cross-sectional HRTEM image of the sample with 50-nm antimony deposited on WSe_2_ surfaces at 120 °C by using thermal evaporation.

In one previous publication, it was demonstrated that single-crystal multilayer antimonene can be grown on MoS_2_ surfaces by using molecular beam epitaxy (MBE)^19^. Significant contact resistance reduction was observed in that paper. Since the growth mechanisms of thermal evaporation are similar to those of MBE, it is possible to grow multilayer antimonene on the same MoS_2_ surfaces by using a thermal evaporator. To investigate this possibility, a 50-nm antimony film is deposited on MoS_2_ surfaces by using thermal evaporation at 200 °C. The cross-sectional high-resolution transmission electron microscopy (HRTEM) image of the sample is shown in Fig. 4(a). As shown in the figure, similar to the MBE-prepared sample, well-stacked multilayer antimonene is also observed on the MoS_2_ surface by using thermal evaporation^19^. The results have demonstrated that by using a different growth technique of thermal evaporation with a lower vacuum requirement, elemental 2D material antimonene can also be grown on MoS_2_ surfaces. Following similar growth procedures, a 50-nm antimony film is also deposited on the WSe_2_ surface by using thermal evaporation at a reduced growth temperature of 120 °C. The Raman spectrum of the sample is shown in Fig. 4(b). The Raman spectra are measured at the center of the WSe_2_ flake after antimonene growth. Since multilayer antimonene will fully cover the WSe_2_ surface, a similar Raman spectrum will be obtained across the WSe_2_ flake. In addition to the Raman peaks corresponding to WSe_2_, additional peaks are observed at 118 and 153 cm^−1^ after antimony deposition, which correspond to the E_g_ and A_1g_ Raman peaks of antimonene, respectively^19^. The similar Raman peaks to those of the MBE-prepared multilayer antimonene film grown on MoS_2_ surfaces suggest that by using thermal evaporation, antimonene can also be formed on WSe_2_ surfaces. To further investigate the crystalline quality of the antimonene film, the cross-sectional HRTEM image of the sample is shown in Fig. 4(c). As shown in the figure, in addition to monolayer WSe_2_, well-stacked layered multilayer antimonene is observed. The layer separations of WSe_2_ and antimonene are 0.7 and 0.4 nm, respectively, which are consistent with previous publications^19,20^. The similar well-stacked layered antimonene film grown on both WSe_2_ and MoS_2_ surfaces suggests that van der Waals epitaxy of the same 2D material may occur on different 2D material surfaces. Besides antimonene, in one pervious publication, we have also demonstrated that other group-IV elemental 2D materials can also be grown on MoS_2_ surfaces^21^. Since the lattice constants of these 2D materials are quite different, the results have demonstrated that the van der Waals epitaxy takes place on 2D material surfaces will help on the crystal growth of the epi-layers and is less dependent on the substrate constants. The lower dependence on the substrate lattice constant of the van der Waals epitaxial growth mode may create more possibilities for crystal growth on 2D material surfaces.

### Conducting 2D materials as the contact metal

**Figure 5:**
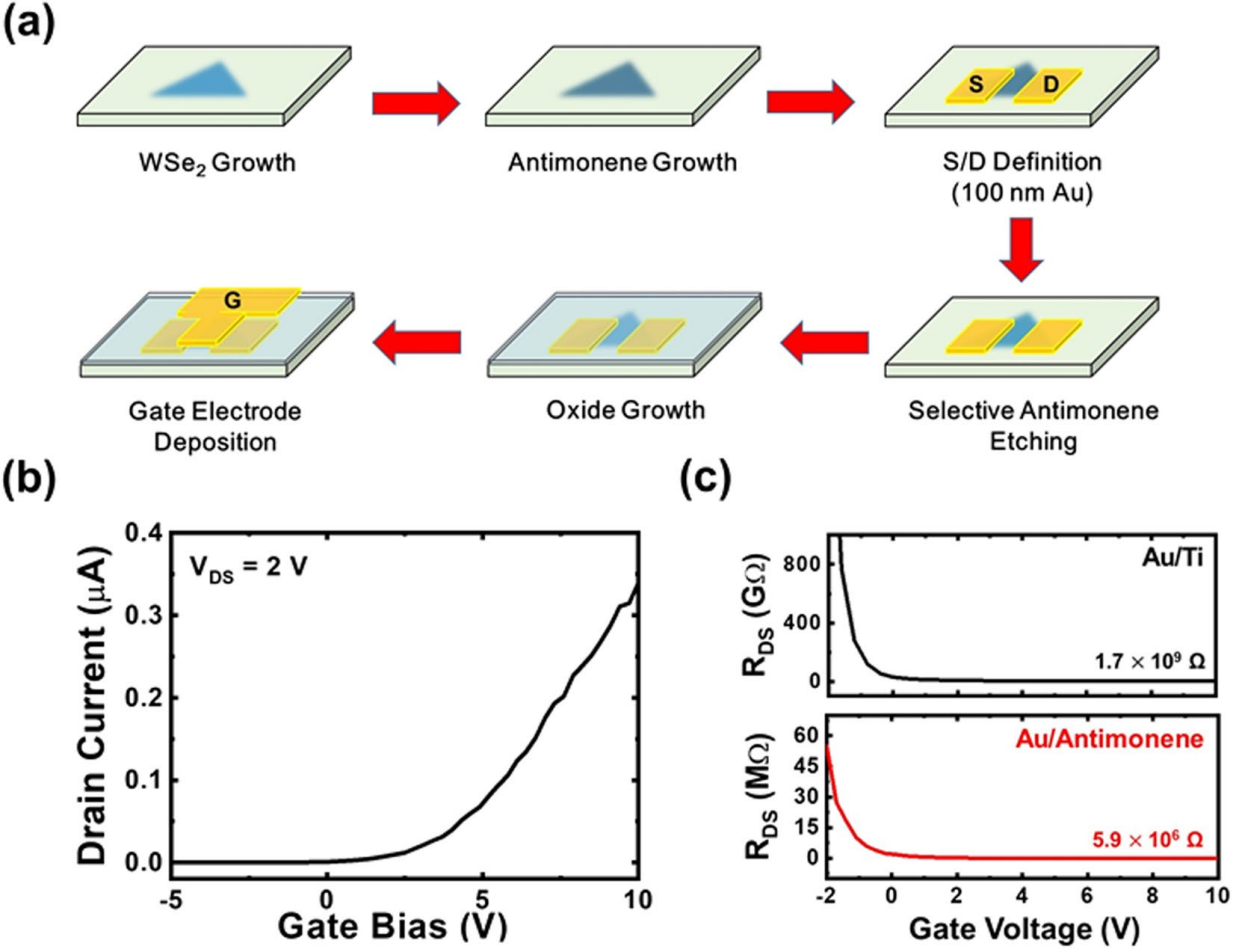
(**a**) The fabrication procedure and (**b**) the I_D_-V_GS_ curve of the WSe_2_ top-gate transistors with Au/multilayer antimonene electrodes. (**c**) The R_DS_-V_GS_ curves of the WSe_2_ top-gate transistors with Au/Ti electrode and Au/multilayer antimonene electrodes, respectively.

The device fabrication procedure for the top-gate WSe_2_ transistor with Au/multilayer antimonene electrodes is shown in Fig. 5(a). After WSe_2_ growth, 100-nm multilayer antimonene is deposited on the sample at 120 °C by using thermal evaporation. After that, 80 × 80 μm^2^ S/D electrodes with 100-nm Au are fabricated on the multilayer antimonene surface following standard photolithography, thermal evaporation and metal lift-off procedures. By using the Au electrodes as the hard mask, the multilayer antimonene outside the electrodes is selectively etched off by dipping the sample into a basic solution for 300 sec.^19^. The oxide growth and gate electrode deposition procedures are the same as those for the device with Au/Ti electrodes. The I_D_-V_GS_ curve of the device at V_DS_ = 2 V is shown in Fig. 5(b). As shown in the figure, compared with the device with Au/Ti electrodes, a significant drain current increase is observed for the device with Au/multilayer antimonene electrodes. The ON/OFF ratio also increases to 4 × 10^4^. The results demonstrate that the high contact resistance between the electrode/2D material interface is indeed one mechanism responsible for the low drain currents of WSe_2_ top-gate transistors. By using multilayer antimonene as the contact metal, the contact resistance is effectively reduced, and higher drain currents can be observed for the device. The increasing ON/OFF ratio of the device also indicates that there is no additional leakage current created between the source and drain electrodes due to the additional multilayer antimonene growth on WSe_2_. The multilayer antimonene can be completely and selectively etched off from the underlying 2D materials. The low gate currents at approximately 10^−11^–10^−12^ A also indicate that the etching procedure does not affect the surface property of WSe_2_ for subsequent Al_2_O_3_ growth. To further investigate this phenomenon, the R_DS_-V_GS_ curves when using Au/titanium and Au/multilayer antimonene electrodes as the contact metal are shown in Fig. 5(c). The R_DS_ values are obtained through the equation R_DS_ = V_DS_/I_D_. Based on the equation R_DS_ = $\frac{{V}_{{DS}}}{{I}_{D}}$ = R_contact_ + R_channel_, R_DS_ has contributions from the contact resistance and the channel resistance. Because the channels of the two devices are all WSe_2_ thin films, the channel resistance should be similar. When the devices turn on, the contact resistance is the major contribution to the R_DS_. In this case, at the same gate voltage, a smaller device R_DS_ indicates a smaller contact resistance. As shown in Fig. 5(c), the R_DS_ values decrease significantly from 1.7 × 10^9^ to 5.9 × 10^6^ Ω at V_GS_ = 10 V, which is similar to the contact resistance reduction of up to two orders of magnitude for multilayer antimonene on MoS_2_ surfaces^19^. The results show that by using a conductive 2D material as the contact metal, electrons can pass through the barrier-free interface when an external voltage is applied to the electrodes^22,23^. However, the actual mechanism responsible for this phenomenon is still unclear. Further investigation is still required in the future.

## Conclusion

In conclusion, we have demonstrated that with a predeposited thin Al_2_O_3_ layer using an e-beam evaporator, a uniform dielectric layer can be obtained on WSe_2_ surfaces after ALD of an additional Al_2_O_3_ layer. By using oxide stacks as the gate dielectric, device performances are observed for WSe_2_ top-gate transistors. With epitaxially grown multilayer antimonene on WSe_2_ surfaces using thermal evaporation as the contact metal, significant increases in the drain currents and ON/OFF ratios are observed. The results demonstrate that the high contact resistance between metal/2D material interfaces is a critical issue for 2D devices. The similar well-stacked layered multilayer antimonene film grown on both WSe_2_ and MoS_2_ surfaces suggests that van der Waals epitaxy of the same 2D material may occur on different 2D material surfaces. The lower dependence on the substrate lattice constant of the van der Waals epitaxial growth mode may create more possibilities for crystal growth on 2D material surfaces. The results have also demonstrated that the stacking of 2D materials with different materials plays an important role in the practical applications of 2D devices.

## Methods

For the preparation of WSe_2_, WO_3_ and pure Se were chosen as the precursors. WO_3_ (0.26 g) was placed in a ceramic boat located in the center of the furnace tube heating zone. During the growth procedure, Se (0.45 g) was placed in a ceramic boat maintained at 250 °C. The sapphire substrate was placed top down facing the WO_3_ precursor in the center of the furnace tube. The Se vapor was transferred to the substrate using an Ar/H_2_ mixture gas as the carrier gas (Ar = 85 sccm, H_2_ = 15 sccm, pressure = 100 Torr). The central heating zone was heated to 950 °C at a ramp rate of 20 °C/min for WSe_2_ growth. After reaching 950 °C, the sample was left for a growth duration of 30 minutes. After growth, the furnace was cooled to room temperature to remove the sample. For the top-gate transistor fabrications, two growth techniques of e-beam evaporation and ALD were adopted for the dielectric layer growth. The growth temperature for Al_2_O_3_ using the e-beam evaporator was 70 °C. After the deposition of 5 nm Al_2_O_3_ by using the e-beam evaporator, the other 20 nm Al_2_O_3_ layer is grown by using the ALD. Before growth, the reaction chamber was pumped down to 1 mTorr. Trimethylaluminum (TMA) and H_2_O vapor were used as the precursor and reactant for aluminum and oxygen, respectively. Each ALD cycles consisted of a 20 ms TMA, a 5 sec. N_2_ purge, a 20 ms H_2_O pulse, and a 5 sec. N_2_ purge. The growth temperature is kept at 180 °C. Devices with Au (100 nm)/Ti (10 nm) and Au (100 nm)/multilayer antimonene (100 nm) electrodes were fabricated using a thermal evaporator and standard photolithography and metal lift-off procedures. For the growth of multilayer antimonene on WSe_2_ surfaces, the same thermal evaporator was adopted. The chamber was pumped down to 3 × 10^−6^ Torr before growth. Antimony flakes were loaded in a tungsten boat as the source. During the deposition procedure, the substrate was kept at 120 °C, and the deposition rate was 0.5 Å/sec. The channel length/width were 5 and 40 μm, respectively, for the devices. The I-V curves for the devices with different electrodes were measured by using a Keithley 2636B system. The Raman and photoluminescence (PL) spectra were obtained using a HORIBA Jobin Yvon HR800UV Raman spectroscopy system equipped with a 488 nm laser. To obtain the surface morphologies, AFM measurements were carried out with a BRUKER Dimension ICON AFM system. The cross-sectional HRTEM images were obtained by using a JEOL JEM-2800F TEM system operated at 200 kV.
